# Ensemble-Based Network Aggregation Improves the Accuracy of Gene Network Reconstruction

**DOI:** 10.1371/journal.pone.0106319

**Published:** 2014-11-12

**Authors:** Rui Zhong, Jeffrey D. Allen, Guanghua Xiao, Yang Xie

**Affiliations:** 1 Quantitative Biomedical Research Center, Department of Clinical Sciences, University of Texas Southwestern Medical Center, Dallas, Texas, United States of America; 2 Harold C. Simmons Comprehensive Cancer Center, University of Texas Southwestern Medical Center, Dallas, Texas, United States of America; Leibniz-Institute for Farm Animal Biology (FBN), Germany

## Abstract

Reverse engineering approaches to constructing gene regulatory networks (GRNs) based on genome-wide mRNA expression data have led to significant biological findings, such as the discovery of novel drug targets. However, the reliability of the reconstructed GRNs needs to be improved. Here, we propose an ensemble-based network aggregation approach to improving the accuracy of network topologies constructed from mRNA expression data. To evaluate the performances of different approaches, we created dozens of simulated networks from combinations of gene-set sizes and sample sizes and also tested our methods on three *Escherichia coli* datasets. We demonstrate that the ensemble-based network aggregation approach can be used to effectively integrate GRNs constructed from different studies – producing more accurate networks. We also apply this approach to building a network from epithelial mesenchymal transition (EMT) signature microarray data and identify hub genes that might be potential drug targets. The R code used to perform all of the analyses is available in an R package entitled “ENA”, accessible on CRAN (http://cran.r-project.org/web/packages/ENA/).

## Introduction

With the advent of high-throughput technologies such as microarrays, next generation sequencing, and other state-of-the-art techniques, huge datasets have been generated in a variety of contexts (*e.g.*, cancer and aging) in order to identify novel biomarkers and drug targets [1]. However, the utility and interpretation of those collected data remains challenging and needs to be improved. Recently, reconstructions of gene regulatory networks (GRNs) from high-throughput data have been widely used to identify novel drug targets or therapeutic compounds [1]–[4]. GRNs provide new information regarding gene-gene interactions and how they work in networks to regulate cellular functions, allowing for a systematic understanding of the molecular and cellular mechanisms underlying specific biological functions and processes [5]–[10]. For GRNs in particular, genes that have many interactions with other genes (called “hub genes”) are likely to be “drivers” of disease status, based on their GRN regulatory roles. An analysis of hub genes is thus a promising approach for identifying key tumorigenic genes for both basic and clinical research [11]–[15].

Although accurate reconstruction of GRNs has proven valuable to a myriad of areas throughout biomedical research, the method remains only moderately satisfactory [7]–[10]. Researchers have previously used approaches such as Bayesian Network- [16], [17], Correlation- [18], and Partial-Correlation-based approaches [19], [20], all of which have demonstrated various strengths and weaknesses under different biological/simulation settings, with no one method excelling under all conditions [21]. Additionally, leveraging gene expression data from multiple datasets to construct gene networks is often difficult, due to discrepancies in microarray platform selection as well as in normalization and data processing techniques [22]–[24]. In this study, we propose an Ensemble-based Network Aggregation (ENA) approach to integrate gene networks derived from different methods and datasets, to improve the accuracy of network inference.

For the construction of our ENA, we used a non-parametric, inverse-rank-product method to combine networks reconstructed from the same set of genes. The rank-product method, introduced by Breitling et al [21], [25], [26], is effective for detecting differentially expressed genes in microarray studies. Because the rank-product method is both powerful and computationally efficient, it has now been extended for use in other fields, such as RNAi screening [27] and proteomics [28]. Additionally, this method can be directly related to linear rank statistics [29]. In this study, we show three ways to leverage this approach to generate ensemble-based networks: 1) samples in a dataset can be “bootstrapped” to reconstruct multiple networks out of a single original dataset using a single reconstruction method, which can then be aggregated into a more accurate and reproducible network; 2) networks produced by various reconstruction methods can be aggregated into a single network that is more accurate than the network provided by any individual method; and 3) networks reconstructed from different studies that contain the same genes can be combined into a single, more accurate network, despite differences in platforms or normalization techniques. Because this approach requires few resources, it can be applied efficiently to dozens or hundreds of networks reconstructed on the same set of genes. We show here that this approach has the ability to improve the accuracy of GRN reconstruction in all three of the above-described applications, based on simulated gene expression data as well as on *Escherichia coli* (*E. coli*) datasets [30]–[33].

An important application of network reconstruction is to identify hub genes in a network that might be biologically and pharmaceutically interesting. When we applied ENA to microarray data that was previously used to delineate an epithelial-mesenchymal transition (EMT) signature [34], we built a network for the identification of hub genes that had been experimentally validated to be EMT-relevant, thus representing potential drug targets. Though our demonstration is focused on microarray data for consistency purposes, ENA should be easily implemented in the analysis pipeline of next-generation sequencing (NGS) data, such as RNA-Seq. Cutting-edge technology enables the simultaneous measurement of millions of cellular data points and sheds light on a brand-new pattern in drug discovery, where medication is viewed in the context of pathways and networks rather than individual proteins or genes [1]. In the near future, in combination with patient-specific genomic profile and drug-target interaction knowledge, GRNs could be used to facilitate both the prediction and treatment of personalized therapy [2].

## Materials and Methods

### Overview of the inverse-rank-product network aggregation approach

Reconstructed gene networks are often returned as a weighted undirected graph 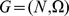, where 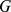 is a reconstructed graph, 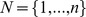 is the set of vertices (genes) in the graph, and 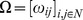 is referred to as the adjacency matrix, in which 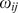 represents the confidence score of the interaction between genes i and j. A larger (absolute) value of 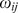 indicates a stronger interaction or higher confidence in the edge between genes i and j, while 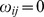 indicates no interaction or conditional independence between genes i and j. Some techniques, such as Sparse PArtial Correlation Estimation (SPACE) [19], return a sparse matrix in which many of the possible interactions are 0; other techniques return complete graphs in which all edges are assigned non-zero weightings. Additionally, the distribution of 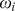 can vary drastically among reconstruction techniques. For this reason, aggregating networks that were reconstructed using different techniques or different datasets is challenging. However, the rank-based method offers a non-parametric approach that does not depend on the actual distribution of scores of edges derived from different methods [35]. In this study, we used a rank-product method to combine networks to overcome the problem of different distributions observed in this approach.

Specifically, suppose 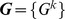 is a set of networks constructed on the same set of genes N, where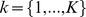 is the index of a particular network. For each single network 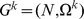, we calculate 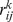, the rank of 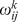 for 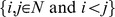. Since the adjacency matrix Ω of an undirected graph is symmetric, we only need to calculate the rank of the 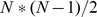 elements in 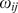, constituting the lower triangle (i<j) of Ω. In this study, we assign the lower rank to the higher confidence interaction. For example, the interaction with the highest confidence will have rank 1. This operation is performed on each individual graph 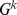 independently. After the rank of 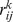 has been computed for each network 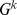, we calculate the rank of a particular edge between genes i and j in the aggregated network by taking the product of the ranks of the same edge across all networks in 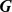, according to: 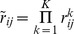. This function is iterated over all possible edges to construct the aggregated network 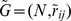, in which the confidence scores of the edges in the new network are based on the aforementioned rank-product calculation.

This algorithm can be efficiently applied to large networks with many reconstructed networks in 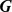. The complexity of the algorithm is that 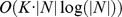, as 
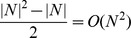
 elements must be sorted for each network in 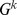.

### Three applications of our ENA approach

The initial application was to leverage the rank-product method to “bootstrap” samples. Each time, we constructed the gene network using a randomly selected subset of the available samples. By repeating this process B times, we created a set 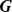 consisting of B graphs, each reconstructed using only randomly selected bootstrap samples in the dataset. For example, here is the procedure to generate the bootstrapping network from a microarray dataset designated MD:
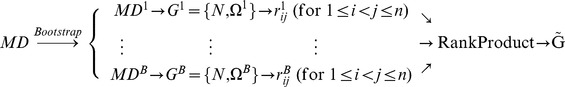



Of course, this bootstrapping procedure inflates the computational complexity of GRN reconstruction by several orders of magnitude, as GRNs must be reconstructed B times rather than just once. Because each graph in 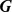 can be reconstructed independently, it is possible to take advantage of the “parallelizability” of these simulations by utilizing multiple cores or computers, as we discuss below. Note also that the complexity of GRN reconstruction does scale on the order of samples included, so that each permuted GRN can be constructed slightly more quickly than a single global GRN. For the reconstruction techniques employed in this study, however, the performance did not vary greatly based on the number of samples included.

The second application of the rank-product network merging method was to reconstruct an aggregated GRN, based on the output of multiple different reconstruction techniques. We have observed that reconstruction techniques perform differently based on different simulation settings [21], with no one method outperforming the others on all metrics. Thus, we were interested to see whether or not merging these GRNs would improve performance. In this application, the set of graphs 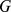
consist of one graph per network reconstruction technique employed. In our analysis, we leveraged GeneNet [20], Weighted Correlation Network Analysis (WGCNA) [18], and SPACE, creating a set of three graphs which could then be aggregated. GeneNet and SPACE are partial-correlation-based inference algorithms. GeneNet uses the Moore-Penrose pseudoinverse [36] and bootstrapping to estimate the concentration matrix. The SPACE algorithm creates a regression problem when trying to estimate the concentration matrix and then optimizes the results with a symmetric constraint and an L1 penalization, while WGCNA is a correlation-based approach that can identify sub-networks using hierarchical clustering. Conceptually, the aggregated graph should place higher confidence on those edges that consistently rank highly across the three methods and lower confidence on those edges that ranked highly in only one graph. The following procedure is used to derive the ensemble network, based on M different methods within the same dataset MD:
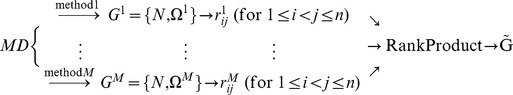



The final application evaluated in this study was in the merging of networks constructed from different datasets. Historically, gene expression datasets have been collected from various sites on different microarray platforms with different procedures for tissue collection, which creates incompatibilities and difficulties when performing analyses on data from different datasets simultaneously. Because the rank-product method makes no assumptions about the distribution of the data at any point, we employ it to combine GRNs produced from different datasets, yielding a single aggregated GRN which aims to capture the consistencies in network topology from the GRNs produced on different datasets. We thus derive the aggregated network from datasets MD^1^, MD^2^…. MD^D^ as follows:
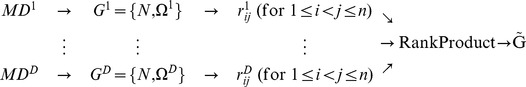



### Software

The code used to bootstrap samples and aggregate the resultant networks was written in the R programming language. We created an R Package entitled “ENA” and made it available on CRAN (http://cran.r-project.org/web/packages/ENA/index.html), from which the compiled binaries, as well as all original source code, are also available for download.

Because of the parallelization opportunities in this algorithm, we ensured that our software would be able to distribute the bootstrapping process across multiple cores and multiple nodes using MPI [37]. Thus, if 150 CPU cores were available simultaneously, a bootstrapping of 150 samples could run in approximately the same amount of wall-clock time as a single reconstruction using all the samples. The ENA package includes robust documentation and (optionally) leverages the RMPI package to allow for parallel execution of the bootstrapping simulations, where such a computational infrastructure is available.

Additionally, we leveraged the Git revision control system via GitHub (http://github.com) to control not only the R code developed for the ENA package, but also all code, reports, and data used in the aforementioned simulations and reconstruction techniques; all of this code is freely available at https://github.com/QBRC/ENA-Research. All the data analysis code used to generate the results in this study was compiled into a single report and can be reproduced easily using the knitr R package [38], [39]. Due to the computational complexity involved in reconstructing this quantity of gene regulatory networks, the execution may take some time to analyze larger networks if the process is not distributed across a large computing cluster.

### Reproducibility

Our analysis code and results were structured in reproducible reports, which are publicly available at https://github.com/QBRC/ENA-Research. The results in this study can be regenerated by a simple mouse click to make everything transparent to researchers.

## Results

### Simulation

We first tested the ENA methods on a wide array of simulated datasets. We simulated the gene expression datasets based on previously observed protein-protein interaction networks [40], [41] from the human protein reference database (HPRD), while the expression data were simulated from conditional normal distributions [42]. We extracted five different network sizes in an approximately scale-free topology: 17 genes with 20 connections, 44 genes with 57 connections, 83 genes with 114 connections, 231 genes with 311 connections, or 612 genes with 911 connections by varying the number of publications required for each connection. For example, if we required each connection to be supported by at least 7 publications (the most reliable connections), it resulted in a very small network with 17 connections; while if we required each connection to be supported by at least 3 publications, it led to a very large network with 911 connections. For each network size, we simulated datasets with differing numbers of samples (microarrays): 20, 50, 100, 200, 500, and 1,000. Finally, we varied the noise by setting the standard deviation of the expression values to 0.25, 0.5, 1.0, or 1.5. In total, we generated 120 datasets to cover all possible arrangements of the above variables.

To test the effect of integrating networks derived from different datasets, we generated three different datasets of 200 samples each from the 231-gene networks with noise values (standard deviation of the distribution of gene expression) of 0.25, 1, and 2. We used the methods described above to reconstruct three networks (one from each dataset) and then aggregated those networks. For comparison, we also combined all three datasets into a single dataset containing these 600 samples and then reconstructed a single network from this larger dataset.

The performance of methods in this setting can be represented by a Receiver Operating Characteristic (ROC) Curve, which plots the True Positive Rate against the False Positive Rate, demonstrating the performance of the method at all relevant edge confidence score thresholds. The performance of a method can be quantified by calculating the Area Under the ROC Curve (AUC). The greater the AUC, the better the performance of the method represented. A perfect reconstruction would have an AUC of 1, while a random guess would obtain an AUC of 0.5. An alternative approach to evaluating gene regulatory network reconstruction is the Area Under the Precision Recall curve (AUPR). In a precision recall curve, recall (also known as sensitivity) is plotted against precision (positive predictive value).

### ENA of bootstrapping samples

**Figure 1:**
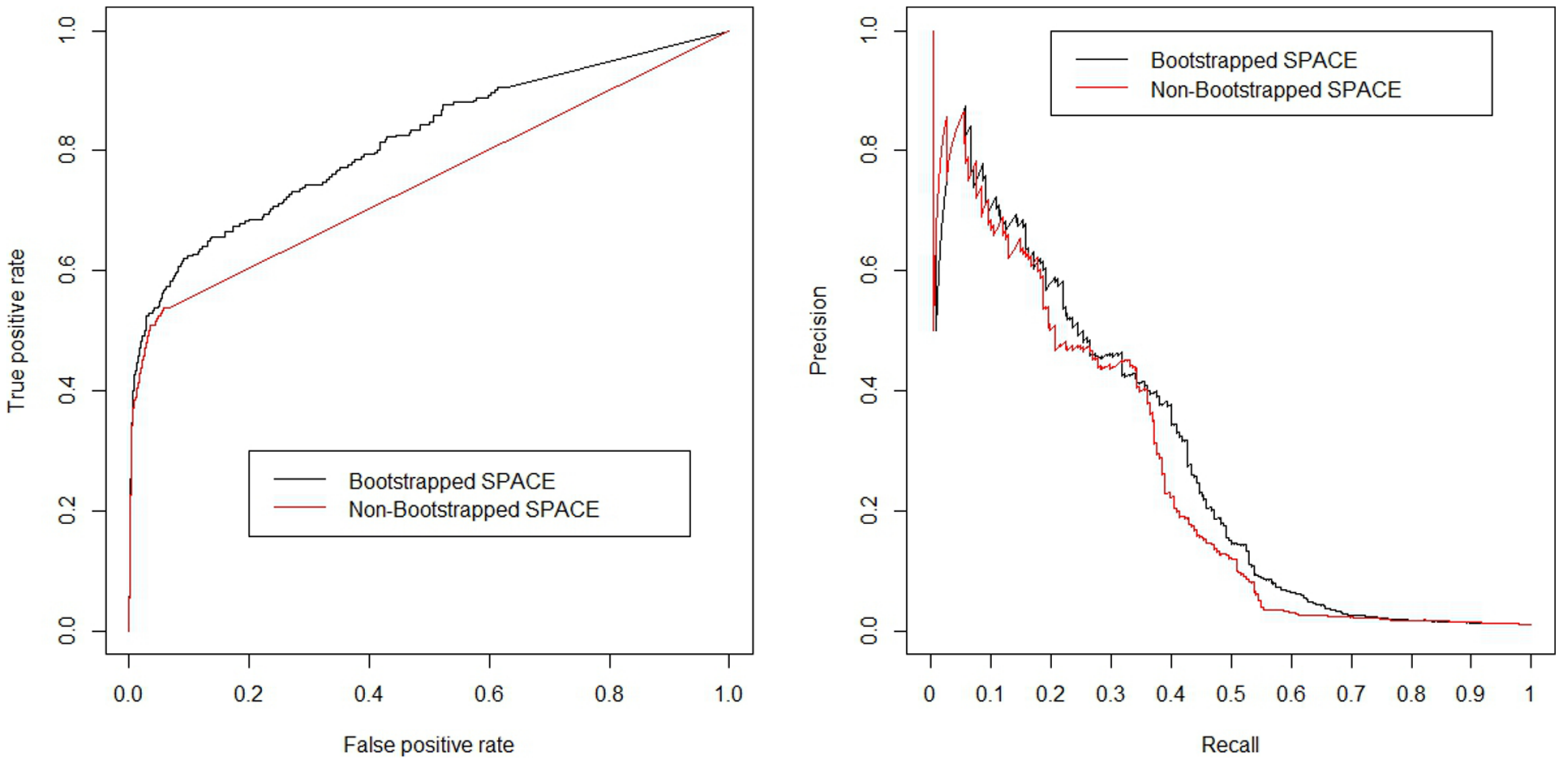
Receiver Operating Characteristic (ROC) curves and the Precision Recall Curve both demonstrate the performance of the SPACE algorithm on the 231-gene network with 20 samples and a noise value of 0.25 when performing a single iteration (*i.e.*, “non-bootstrapped”) or bootstrapping the dataset using the Ensemble Network Aggregation approach. In this case, the Area Under the ROC Curve (AUC) of the non-bootstrapped SPACE method was 0.748, while that of the bootstrapped SPACE method was 0.816. The Area Under the Precision-Recall (AUPR) curve also improves from 0.249 (SPACE) to 0.273 (bootstrapping).

**Figure 2:**
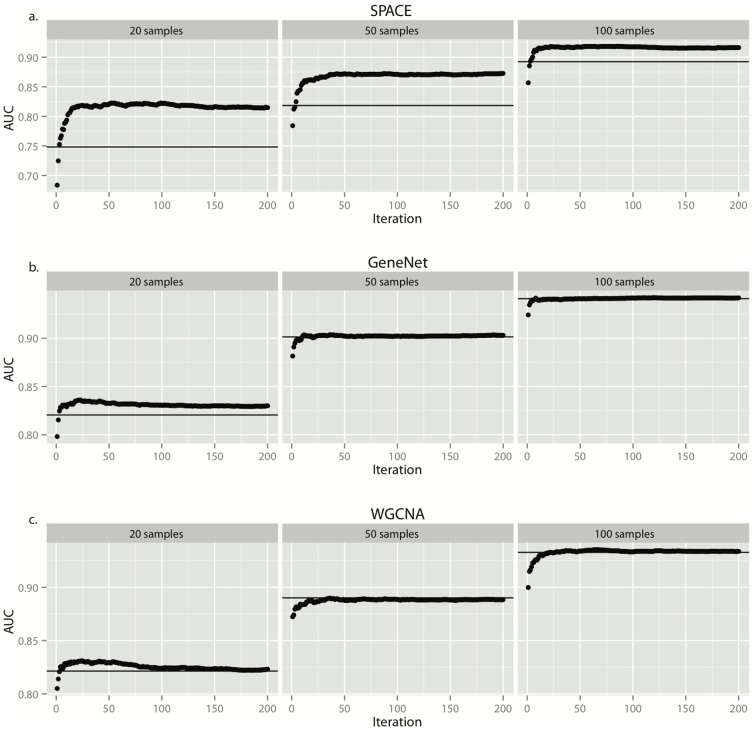
Comparison of the Area Under the Curves (AUCs) of the re-constructed networks from the 231-gene network with a noise value of 0.25 and different sample sizes (20, 50 or 100) for SPACE (a.), GeneNet (b.), and WGCNA (c.). In these plots, the y-axis shows the performance of the reconstructed network, measured by the AUCs; a horizontal line is drawn to represent the AUC of the non-bootstrapped reconstruction (a single reconstruction using all available samples). The x-axis represents the number of iterations in the bootstrapping process. Points below the horizontal line represent a loss in accuracy of the reconstructed networks, and points above the horizontal line represent a gain of AUC (*i.e.*, an increase in model performance).

We found that bootstrapping samples can increase the accuracy of network inference. In our study, we randomly selected 70% of all samples and rebuilt networks and repeated the abovementioned process more than 100 times for each dataset to get the bootstrapping results. For example, the networks reconstructed from the dataset on the 231-gene network with a noise value of 0.25 can be compared to demonstrate variations in performance (Figures 1 and 2).

**Figure 3:**
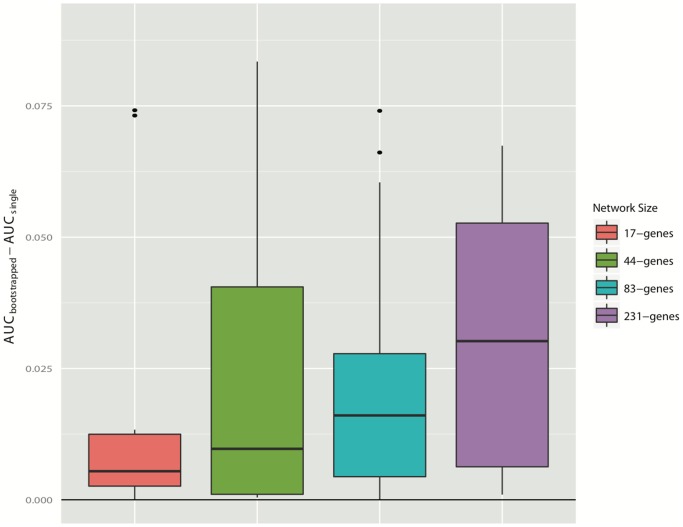
The effect of network size on ENA performance. The y-axis represents the improvement in AUC of the bootstrapped SPACE networks vs. the non-bootstrapped SPACE networks. Different bars represent different sizes of networks in the simulation study.

Figure 1 (left) shows that by bootstrapping samples using the SPACE algorithm, the AUC of the reconstructed network can improve from 0.748 to 0.816. In order to evaluate the precision of ENA, we also plotted the Precision-Recall Curve (Figure 1, right); the area under the precision-recall curve improved from 0.249 to 0.273. Figure 2 shows the degree of AUC improvement with each iteration of bootstrapping in SPACE, WGCNA and GeneNet with sample sizes of 20, 50 and 100 (left, middle and right panels). As shown in this figure, the bootstrapping method increases the performance of SPACE substantially, improves GeneNet slightly (when the number of microarrays is small), but does not noticeably improve the performance of WGCNA. The AUC improvements for different sample sizes and different network sizes are plotted in Figures S1–S4 in File S1. From these figures, we can see that SPACE benefits from bootstrapping in 80% of all simulated networks and in 89% of “large” network simulations. Figure 3 shows the average performance increase achieved by bootstrapping SPACE on different network sizes. The improvement increases as the network size increases. Based on this evidence, we suggest employing the bootstrapping approach when using the SPACE algorithm, but not when using the others evaluated in this study.

### ENA of different methods

**Figure 4:**
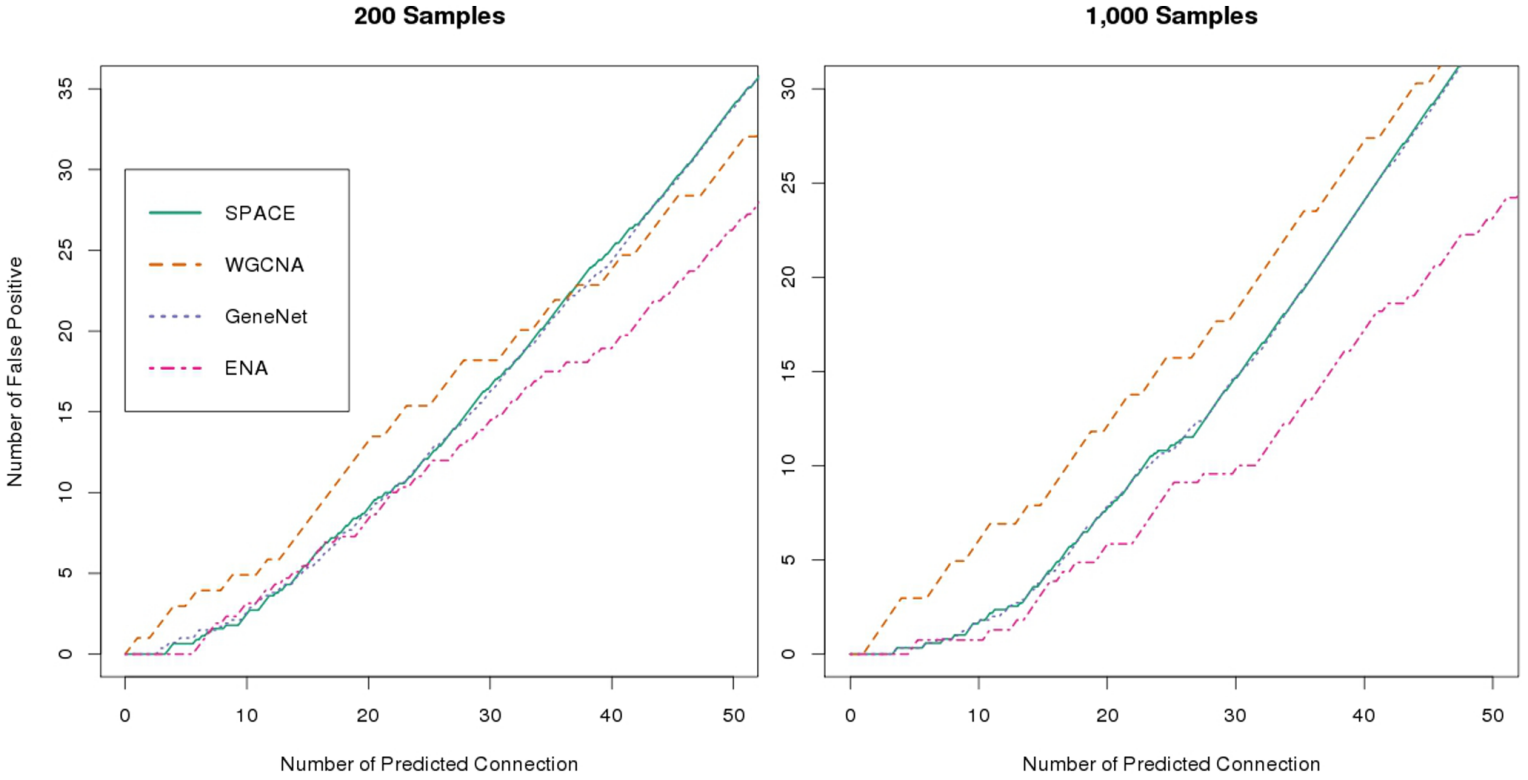
The performance in aggregating different methods. A comparison of the accuracy of the reconstructed networks using the datasets containing 200 samples (left) and 1,000 samples (right) from the 83-gene network with a noise value of 0.25. As can be seen here, the ensemble network aggregation approach performs better than any of the other individual techniques on these two networks.

Aside from optimizing individual reconstruction techniques, we found that combining different network reconstruction techniques that were executed on the same dataset also has the power to significantly improve the accuracy of the reconstructed networks. Using the dataset from the 83-gene network with 200 samples and a noise value of 0.25, we evaluated the comparative performance of each reconstruction technique, as well as that of the aggregated network. Figure 4 shows that the aggregated network outperformed all of the individual constituent reconstruction techniques.

We also observed this trend to hold true across most of the datasets (Figure S5 and Figure S6 in File S1) that we tested: the aggregated method typically outperformed any single reconstruction technique. This is especially beneficial in scenarios in which the top-performing individual network reconstruction technique may vary based on the context, *e.g.*, some methods perform well on larger networks, while others excel in datasets containing few samples. Thus, to have an aggregation technique that consistently outperforms or matches the best performing individual method eliminates the need to choose a single reconstruction technique based on the context.

In addition, we compared our method with the method used in Marbach et al. The result (Figure S8 in File S1) indicates the proposed ENA method performs better in the simulation settings.

### ENA of different datasets

**Figure 5:**
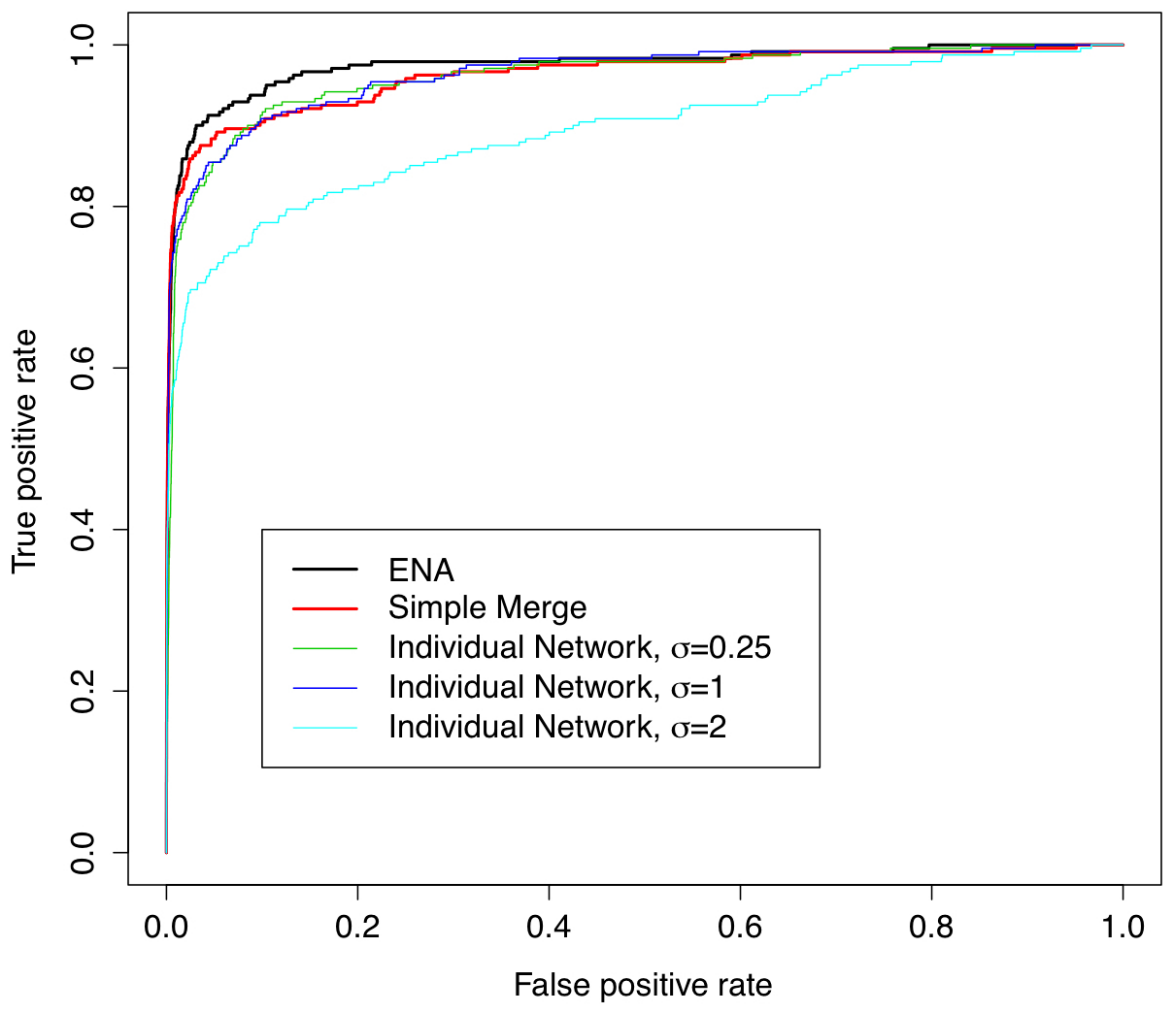
The ROC curves of different approaches to reconstruct the gene network based on three simulated datasets. The ENA approach outperformed the alternative approach of simply combining the expression into a single dataset and individual network with increasing noise of 0.25, 1, and 2. AUCs of all five approaches are 0.98, 0.96, 0.96, 0.96, and 0.89 respectively.

Finally, we found the ENA approach to work very well when attempting to integrate various datasets, especially among heterogeneous datasets containing different distributions of expression data. After generating three datasets from the 231-gene network, each with 200 samples and noise values of 0.25, 1, and 2, we reconstructed each network using bootstrapped SPACE, GeneNet, and WGCNA, and then aggregated the resultant networks into a single network for each of the three datasets. We then used the ENA approach to consolidate these three networks into a single network representing the underlying network behind the three distinct datasets. We also compared this approach to the alternative of simply merging all three datasets into a single 600-sample dataset and using the same approach to reconstruct a single network. As shown in Figure 5, the proposed ENA approach outperformed the alternative approach of simply combining the expression data into a single dataset. Reconstructing on each dataset independently produced AUCs of 0.96, 0.96, and 0.89 from noise values of 0.25, 1, and 2, respectively. “Naïvely” merging the datasets by combining them into one large dataset yielded an AUC of 0.96. The network aggregation approach, however, yielded the best performance, with an AUC of 0.98.

### Evaluating ENA approach in *E. coli* datasets

We then tested the ENA approach on three *Escherichia coli* (*E. coli*) datasets: 1) the Many Microbe Microarrays Database (“M3D”) [30] containing 907 microarrays measured under 466 experimental conditions using Affymetrix GeneChip *E. coli* Genome arrays; 2) the second dataset (“Str”) of expression data from laboratory evolution of *E. coli* on lactate or glycerol (GSE33147) [31], which contains 96 microarrays measured under laboratory adaptive evolution experiments using Affymetrix E. coli Antisense Genome Arrays; and 3) the third dataset [32], [33] (“BC”) containing 217 arrays measuring the transcriptional response of *E. coli* to different perturbations and stresses, such as drug treatments, UV treatments and heat shock. The RegulonDB database [43], [44], which contains the largest and best-known information on transcriptional regulation in *E. coli*, was thus used as a “gold standard” to evaluate the accuracy of the variously constructed networks.

**Figure 6:**
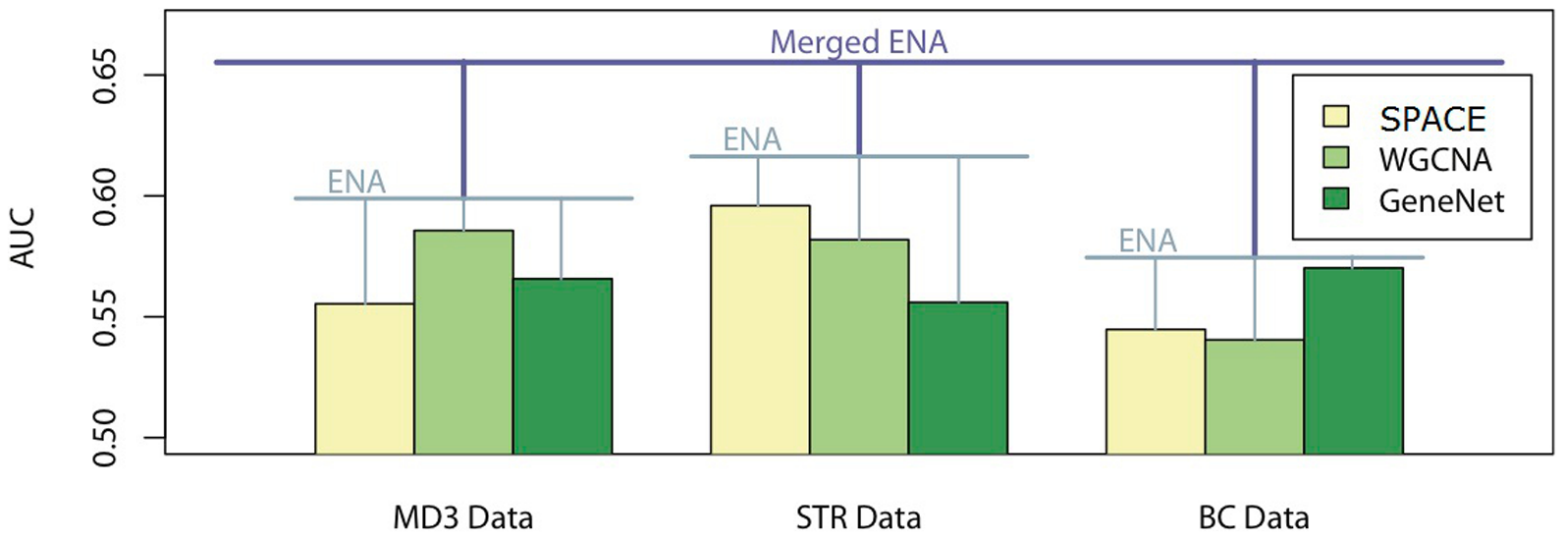
The AUCs of the generated networks when executed on the E. coli datasets. Note that the aggregating ENA networks from SPACE, WGCNA and GeneNet increase the accuracy within each individual dataset, and aggregating results from three datasets further increases the accuracy beyond that of any one dataset.

We were able to obtain similarly positive results by employing these approaches on the *E. coli* data (Figure 6). Bootstrapping and aggregating the three methods on each dataset independently produced AUCs of 0.574, 0.616, and 0.599 for the BC, Str, and MD3 datasets respectively. By merging the three networks produced on each dataset using ENA, we were able to produce a network with an AUC of 0.655, larger than the AUC of any network produced by any of the datasets independently. Because the performance of ENA in the real dataset was evaluated based on our current biological knowledge, which may only be a partial truth, the overall network reconstruction accuracy observed in the real dataset was much lower than those in the simulated datasets, where the full truth was known. On the other hand, simulated data might also partially reflect the true situation by simplifying aspects of an over-complicated biological process. However, the ENA approach consistently improved the network reconstruction accuracy in both simulated and real datasets.

### Network reconstruction via ENA to identify potential drug targets

Network reconstruction of gene expression data helps identify hub genes that might be novel drug targets because of their role in engaging multiple molecules, a process that has been used to identify gene sets predictive of benefit for adjuvant chemotherapy in non-small-cell lung cancer [13]. Here we applied ENA to a dataset consisting of 76 genes from 54 non-small-cell lung cancer (NSCLC) cell lines that were previously identified to comprise an epithelial-mesenchymal transition (EMT) “signature” for NSCLC [34]. This signature consisted of genes whose expressions were either positively or negatively correlated with at least 1 of 4 putative EMT markers, including E-cadherin (*CDH1*), vimentin (*VIM*), N-cadherin (*CDH2*) and/or fibronectin 1 (*FN1*), and followed a bimodal distribution pattern across the cell lines [34].

**Figure 7:**
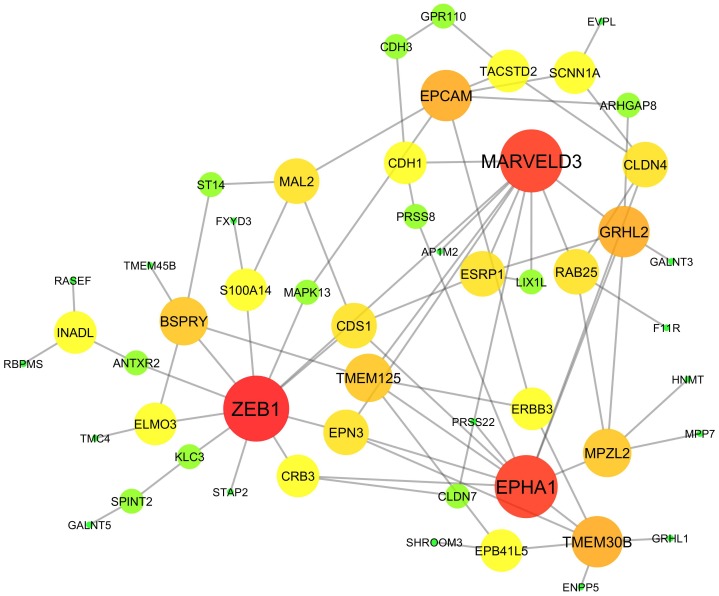
Network reconstruction (based on a previous epithelial-to-mesenchymal transition gene signature) [34] via ENA identifies potential drug targets for non-small-cell lung cancer (NSCLC). Microarray data from 54 NSCLC cell lines were analyzed using four different methods and the results integrated via ENA. Identified hub genes *ZEB1*, *MARVELD3* and *EPHA1* have interesting clinical implications as novel drug targets. Node color and size are proportional to the degree of connectivity (*i.e.*, the number of edges connecting each node).

Overall, we attempted to identify hub genes clinically interesting for NSCLC treatment. We thus employed multiple methods to build GRN networks and combined them via ENA. As shown in Figure 7, we identified three major nodes. Of these, *ZEB1*, which had the highest degree in the resulting ENA network, is a well-known EMT activator and tumor promoter that represses stemness-inhibiting microRNAs [45] and mediates the loss of E-cadherin expression to allow cell detachment [46]. *MARVELD3* is known as a tight junction molecule and has been shown to be downregulated during Snail-induced EMT [47]. Finally, *EPHA1*, the first member of the erythropoietin-producing hepatocellular (Eph) family of receptor tyrosine kinases, was recently shown to potentially play a role in carcinogenesis and the progression of several cancer types [48]. *EPHA1* is also frequently mutated in NSCLC patients, along with other known “driver” mutations [49].

## Discussion

The ability to aggregate networks using the rank-product merging approach has proven to be a valuable contribution in reconstructing gene regulatory networks – and likely in other fields, as well. By bootstrapping a single dataset using a single approach such as SPACE, we were able to significantly improve the performance of the algorithm. By aggregating the networks produced by different reconstruction techniques on a single dataset, we were able to consistently match or outperform the best-performing technique for that dataset, regardless of fluctuations in the performance of any one algorithm. By aggregating networks constructed independently on different datasets capturing similar biological environments, we were able to reconstruct the network more accurately than would be possible using any one dataset alone. So far, the study of integration of gene regulatory networks has been continuously advancing. Both Marbach D. et al. 2012 [50] and Hase T. et al. 2013 [51] have devised methods for integrating gene regulatory networks. The former is based on integration through rescoring gene-gene interaction according to average ranks across multiple methods, while the latter is focused on combining the confidence of each gene-gene interaction by multiple algorithms through leveraging the diversity of the different techniques. ENA is able to integrate networks from multiple algorithms. In addition, ENA performs bootstrapping within single dataset and also takes advantage of integrating multiple datasets to improve the performance. In this study, we showed that when integrating bootstrapped samples, different algorithms and data sets could achieve the best performance (Figure 6).

It is likely that SPACE was the only method to show consistent and significant improvement from bootstrapping because the SPACE algorithm models gene regulation using linear regression; as a result, the network construction problem is converted to a straightforward variable selection problem. In SPACE, the variable selection problem is solved by sparse regression techniques with a symmetric constraint. By solving all the regression models simultaneously, SPACE attempts to accrue the globally optimized results. However, due to the instability in variable selection [52] caused by collinearity in the data, the networks constructed by SPACE are sensitive to sampling. A small change in the samples selected may lead to a relatively large change in the network structure. As a result, the networks constructed from bootstrapping samples are relatively “independent”, which leads to greater accuracy in the aggregated network.

As a sample application, we applied our approach to an EMT signature data set, successfully building a gene regulatory network and identifying hub genes with interesting therapeutic and pharmacological implications (Figure 7). Our discovery has also been experimentally validated in previous literature. Ingenuity Pathway Analysis (IPA) (http://www.ingenuity.com/products/ipa) is a pathway and network database based on curated literatures. When we used IPA to analyze our data, ZEB1 was identified as a hub gene, which confirmed our discovery using the ENA approach. Additionally, predicted interactions such as the CDH1–CDH3 interaction and the CLDN4-GRHL2 interaction were also confirmed (Figure S7 in File S1). While here we showed results only from microarray data analyses, ENA can also be conveniently applied to next-generation sequencing techniques such as RNA-Seq. Thus, combining individualized genomic profiles with the reconstruction of gene regulatory networks might facilitate personalized therapy (possibly using “hub genes” as therapeutic targets).

To make ENA implementation user-friendly for the biological research community, we provide a publically available R package to allow others to use these techniques on their own datasets. By leveraging the MPI framework, we were able to run the bootstrapping process in parallel across many cores and nodes, drastically reducing the amount of time it takes to run such analyses. We include in this package a function that can permute random networks and perform ENA in order to better estimate the significance of any particular connection observed in a network. This function can be used to reduce a continuous, complete graph to an unweighted graph that includes only statistically significant edges.

Finally, we went to great lengths to ensure that all of our analysis would be as reproducible as possible by collating our analysis code into reproducible reports – most of which can be regenerated at the click of a button – and making all of these freely available online at https://github.com/QBRC/ENA-Research. We feel that this transparency is an important but uncommon step in the scientific process and hope that other researchers may begin incorporating such practices into their own investigations to foster more open, collaborative research.

## Supporting Information

File S1 **Supplementary Figures (Figure S1–S8).**
(DOCX)
