# Supplementary material for: Ensemble-Based Network Aggregation Improves the Accuracy of Gene Network Reconstruction
Source: PLoS One. 2014 Nov 12;9(11):e106319. doi: 10.1371/journal.pone.0106319 (PMC4229114; doi:10.1371/journal.pone.0106319)
Supplement: File S1 — Supplementary Figures (Figure S1–S8). (DOCX) [file pone.0106319.s001.docx]

**Ensemble-based network aggregation improves the accuracy of gene network reconstruction**

Rui Zhong^1#^, Jeffrey D. Allen^1, 2#^, Guanghua Xiao^1^, and Yang Xie^1, 2*^

^1^ Quantitative Biomedical Research Center, Department of Clinical Science, ^2^ Harold C. Simmons Comprehensive Cancer Center, University of Texas Southwestern Medical Center, 5323 Harry Hines Blvd, Dallas, TX 75390

# These authors contributed equally to this work

* Correspondence author

Yang Xie, M.D., Ph.D.

Associate Professor

Quantitative Biomedical Research Center

Division of Biostatistics, Department of Clinical Sciences

UT Southwestern Medical Center at Dallas

5323 Harry Hines Blvd, Dallas, TX 75390-8821

Office: 214-648-5178 / Fax: 214-648-1663

Email: Yang.Xie@utsouthwestern.edu


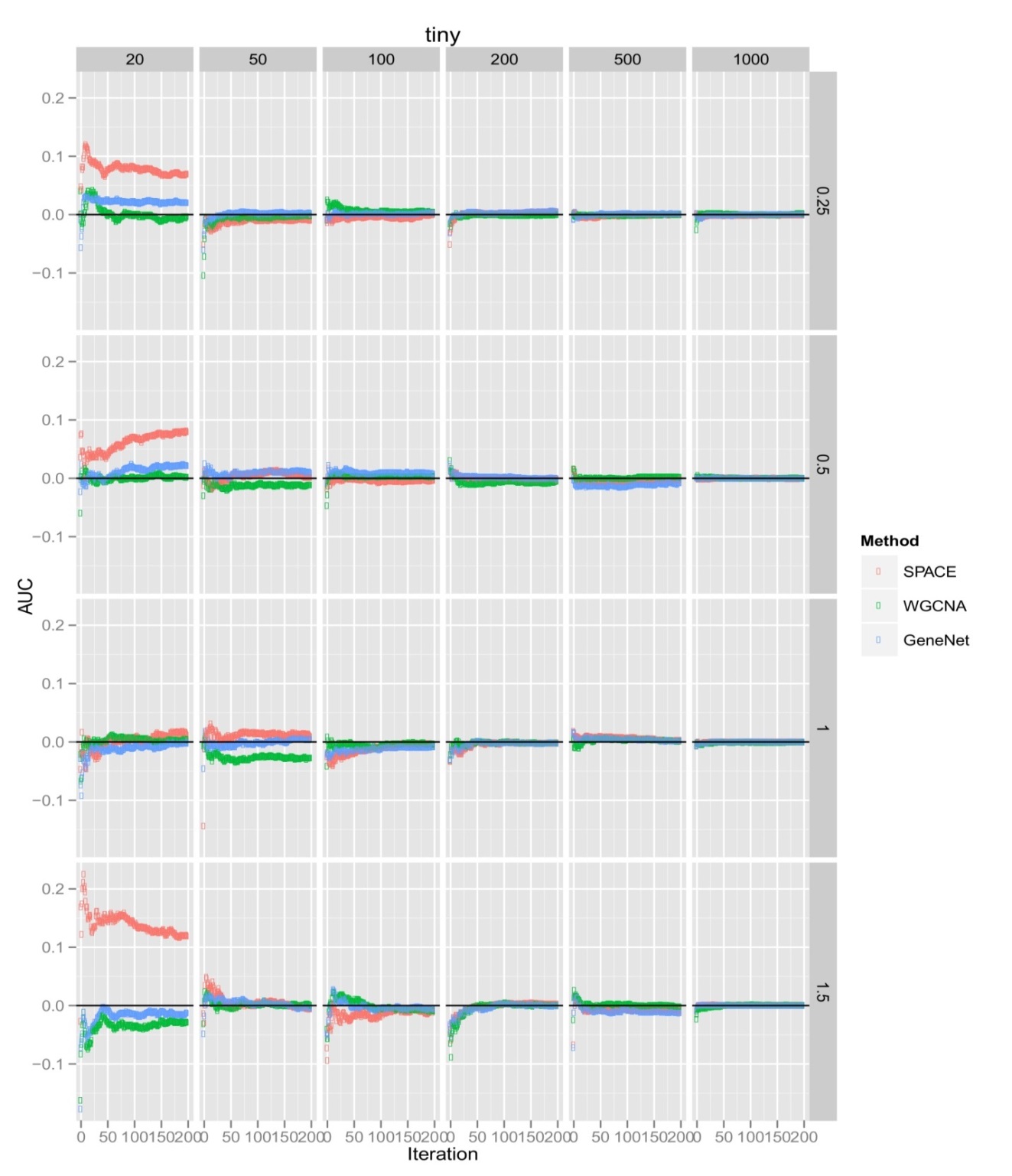


Figure S1. Performance of bootstrapping on simulated dataset of 17-genes network across increasing sample sizes (20, 50, 100, 200, 500, 1000) over increasing iterations (0~200). Improved AUCs were measured and plotted for each iteration.


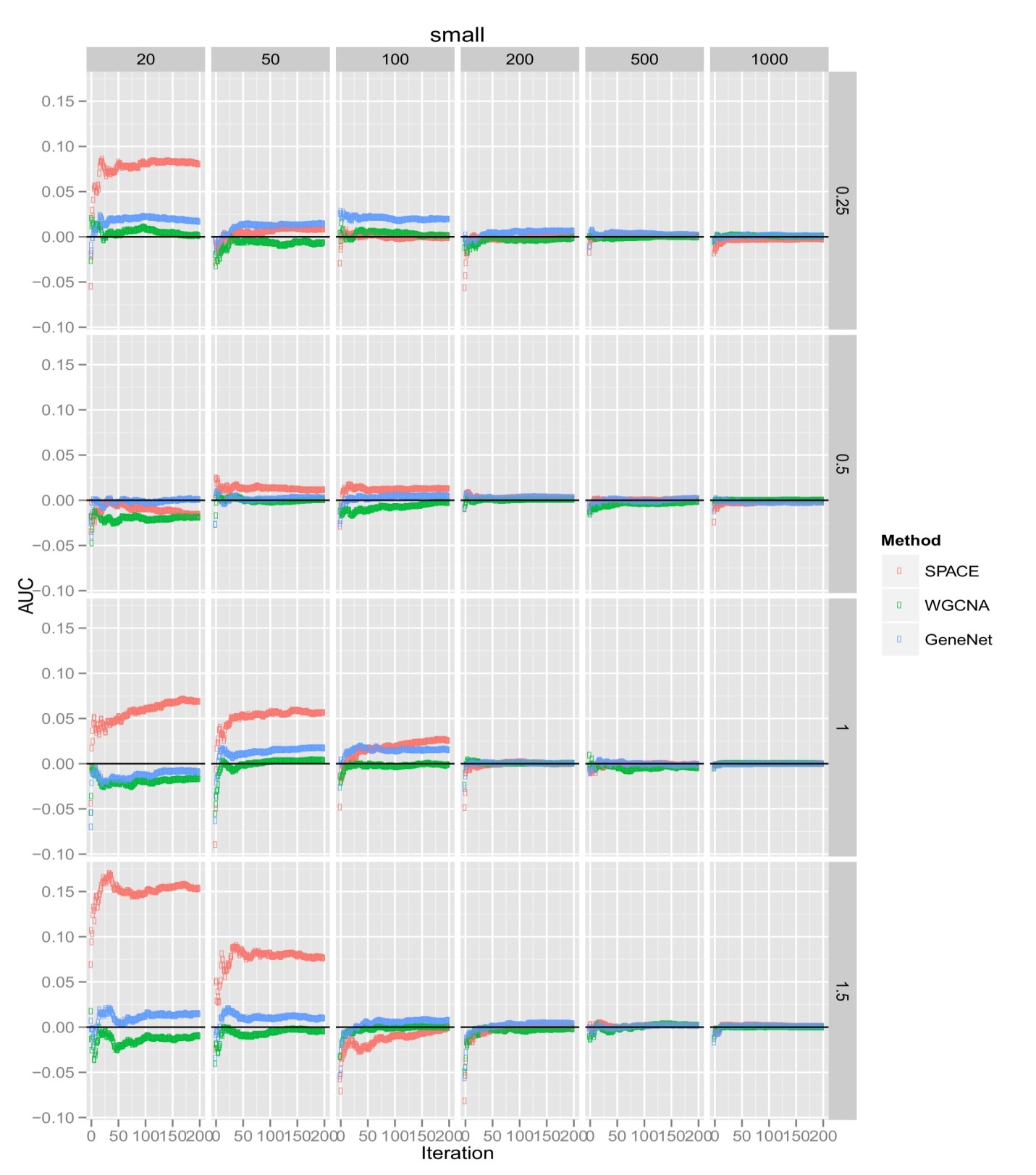
 Figure S2. Performance of bootstrapping on simulated dataset of 44-genes network across increasing sample sizes (20, 50, 100, 200, 500, 1000) over increasing iterations (0~200). Improved AUCs were measured and plotted for each iteration.
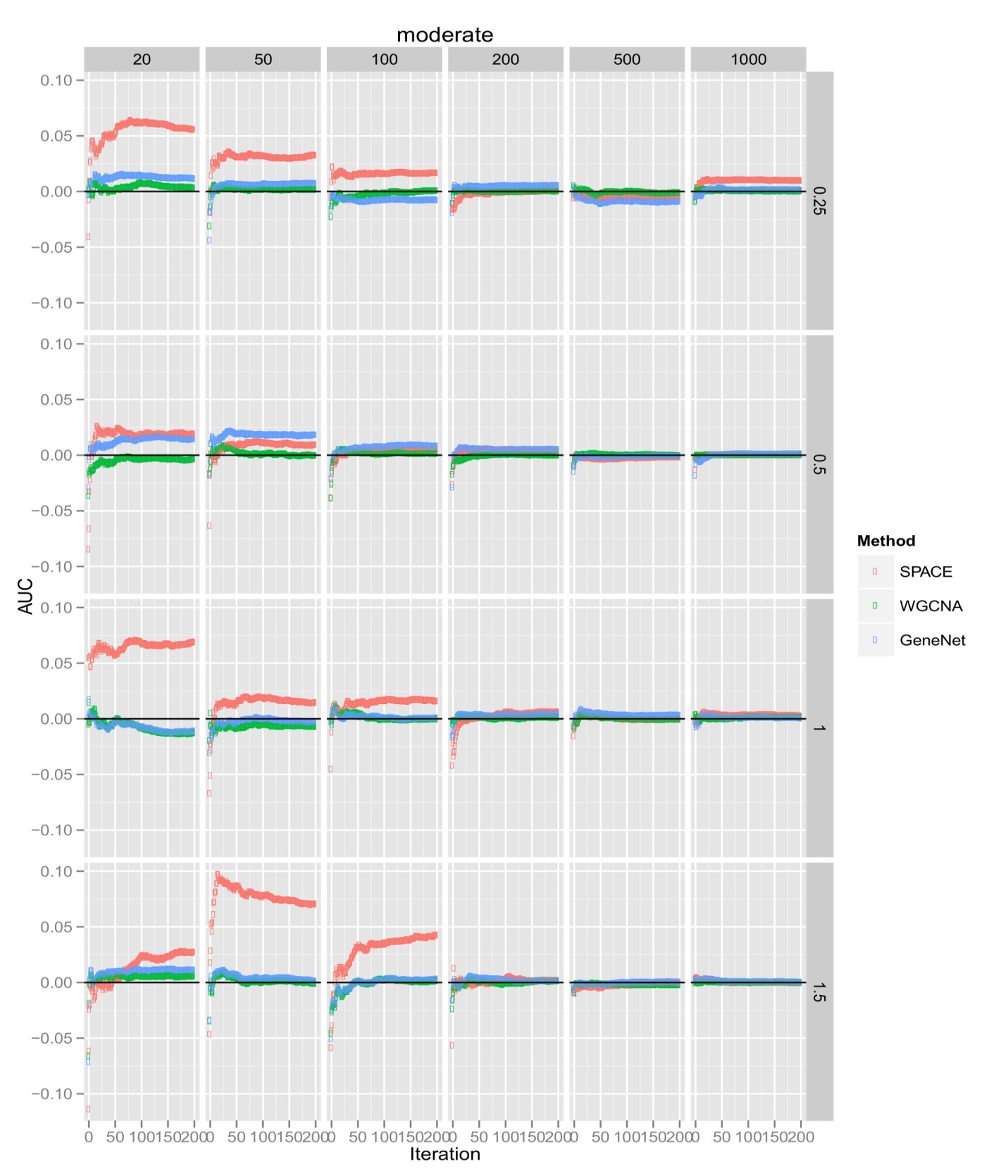
 Figure S3. Performance of bootstrapping on simulated dataset of 83-genes network across increasing sample sizes (20, 50, 100, 200, 500, 1000) over increasing iterations (0~200). Improved AUCs were measured and plotted for each iteration.
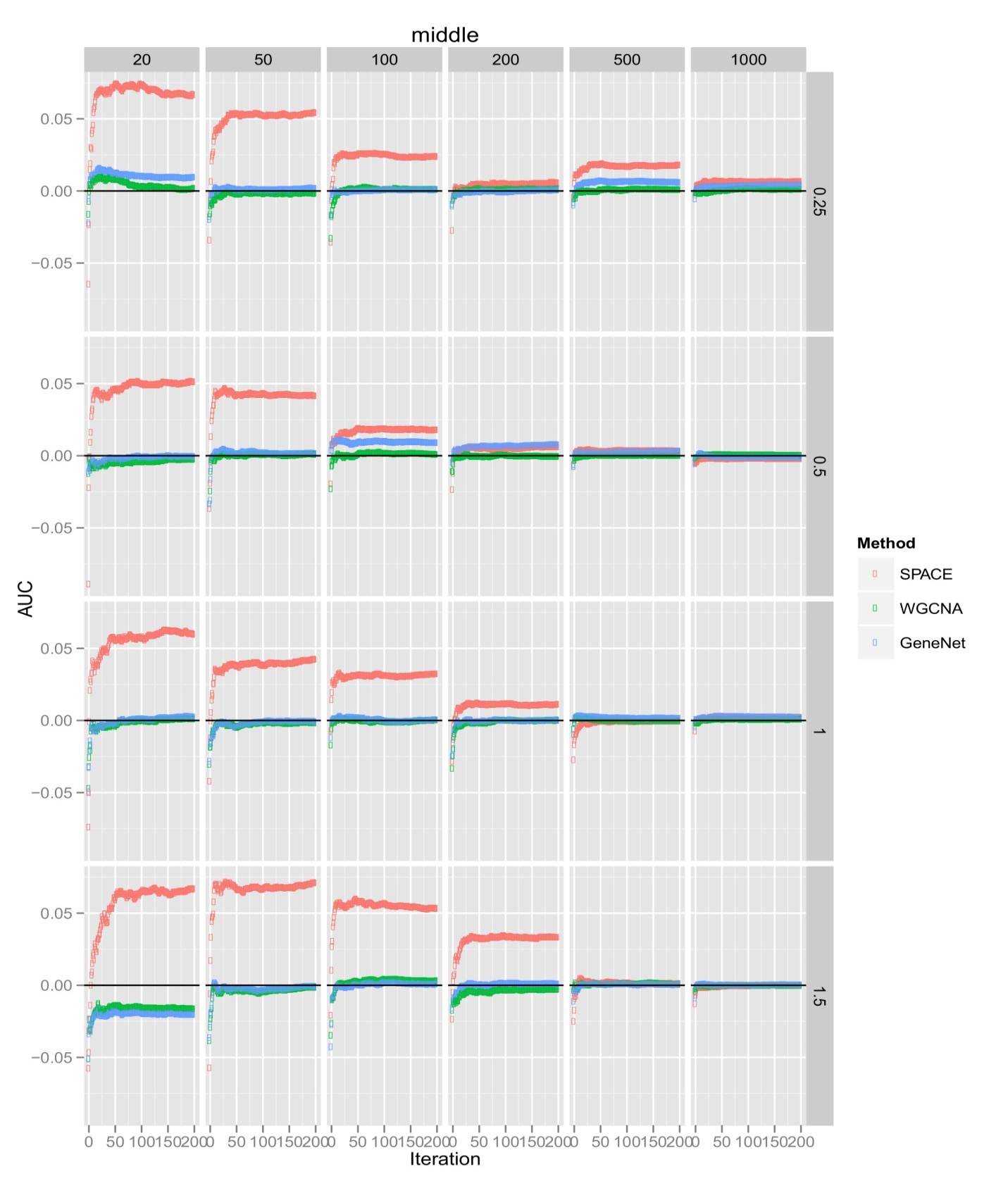
 Figure S4. Performance of bootstrapping on simulated dataset of 231-genes network across increasing sample sizes (20, 50, 100, 200, 500, 1000) over increasing iterations (0~200). Improved AUCs were measured and plotted for each iteration.


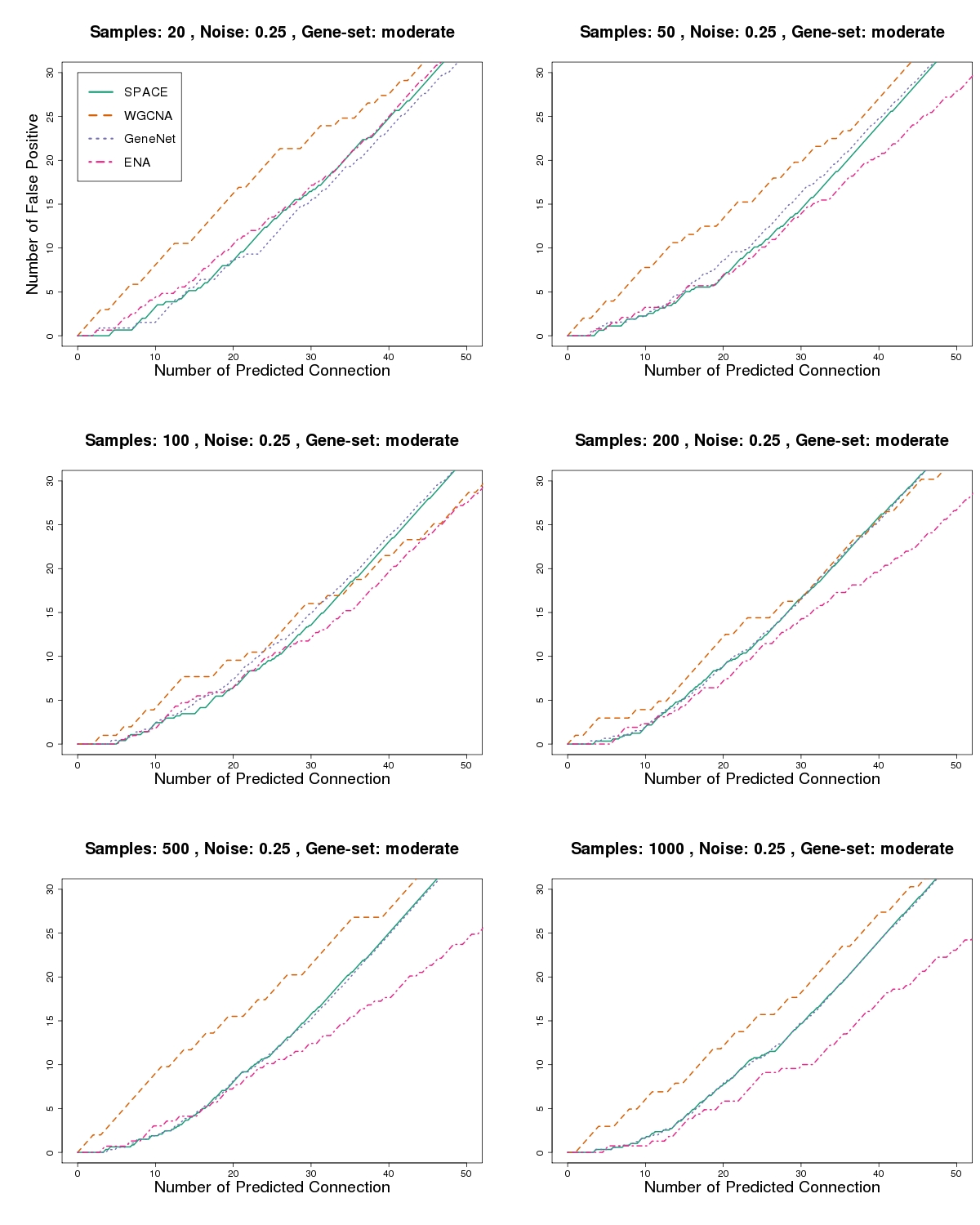


Figure S5. A comparison of the accuracy of the reconstructed networks using the dataset containing samples (20, 50, 100, 200, 500, 1000) from the 83-gene network with a noise value of 0.25.


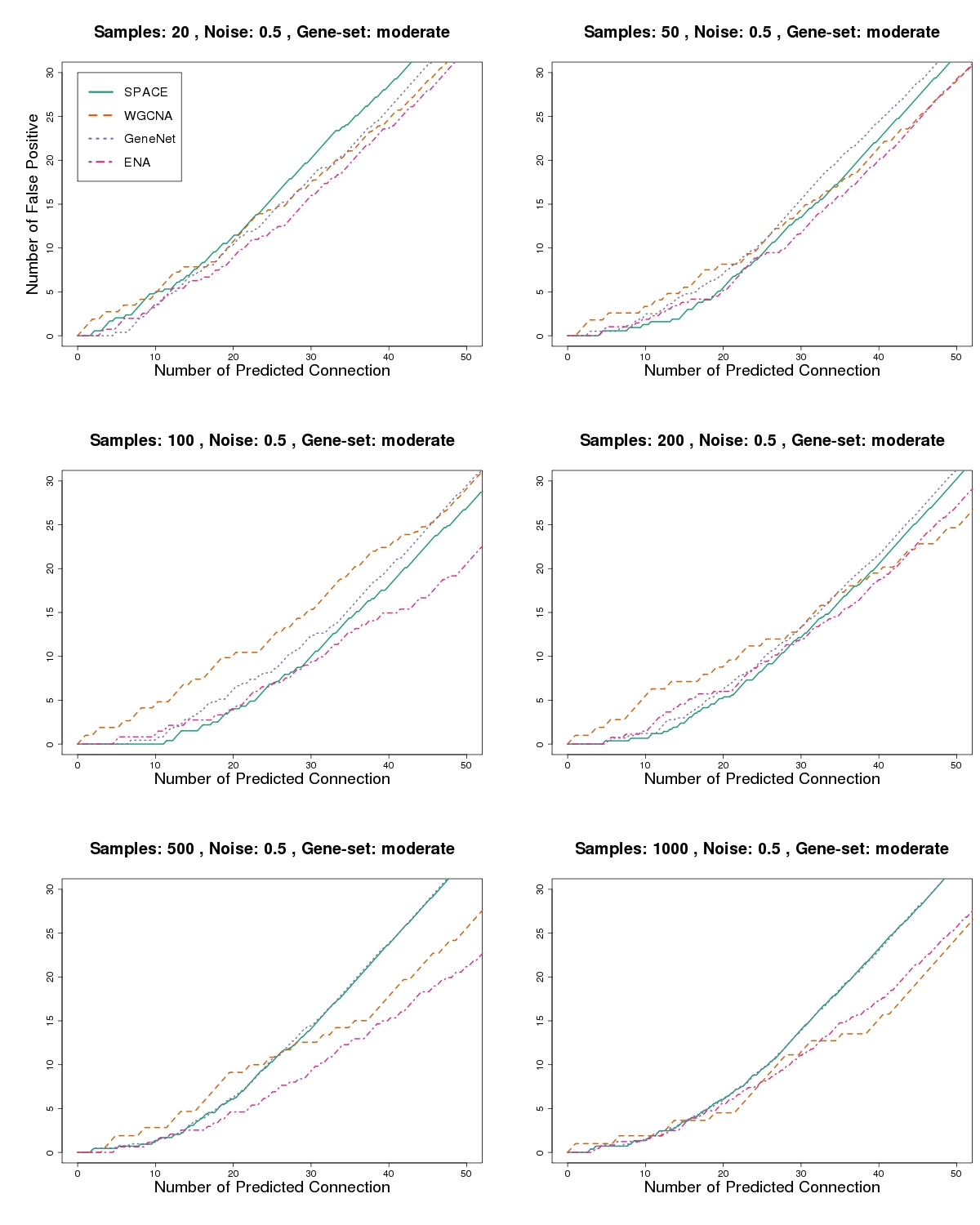


Figure S6. A comparison of the accuracy of the reconstructed networks using the dataset containing samples (20, 50, 100, 200, 500, 1000) from the 83-gene network with a noise value of 0.5.


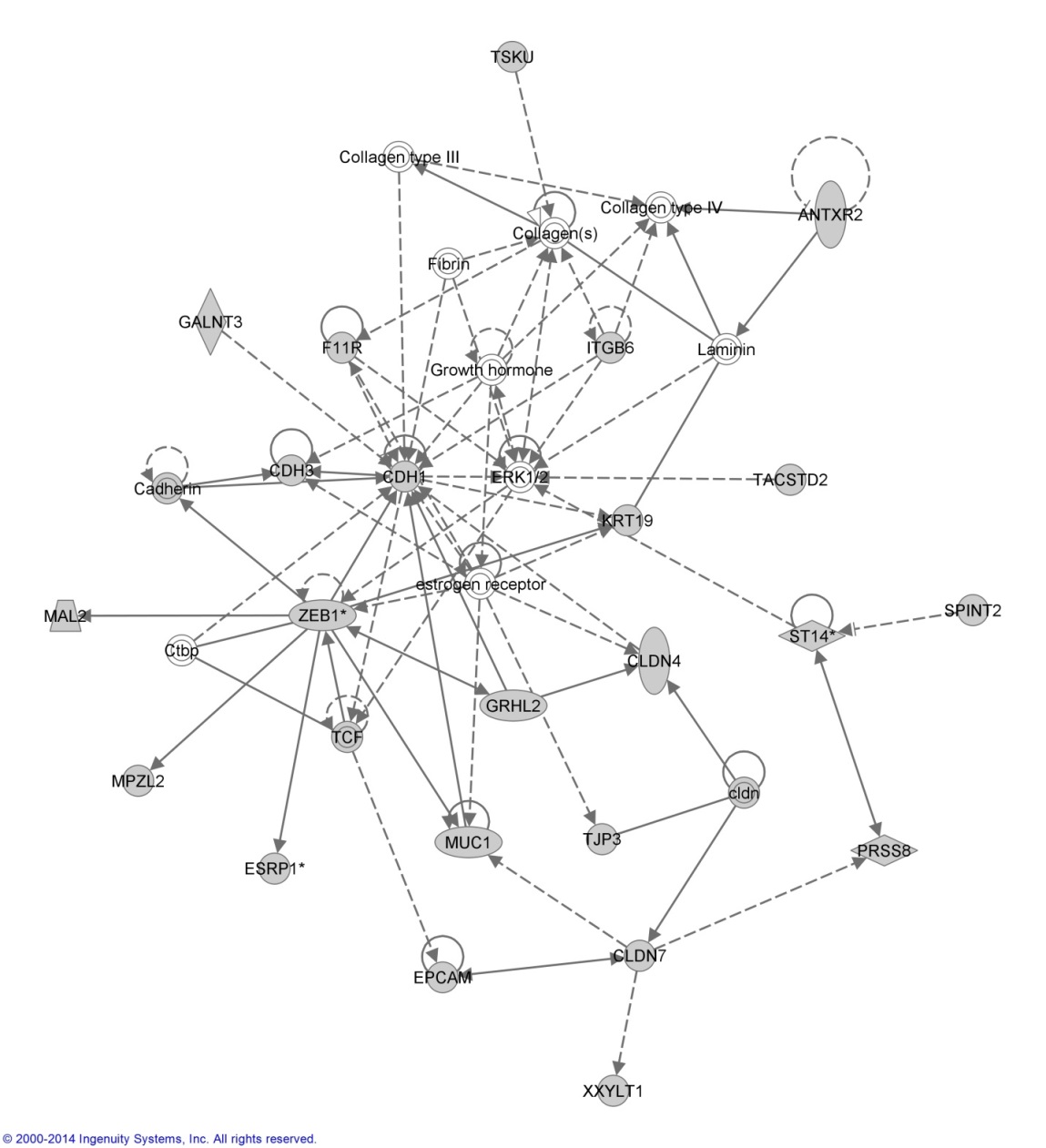


Figure S7. Pathway analysis using Ingenuity Pathway Analysis (IPA) confirmed predicted hub gene ZEB1 played a role in built networks. As well, predicted interactions such as the CDH1-CDH3 interaction and the CLDN4-GRHL2 interaction were also confirmed. Different node shapes represent their genetic functions. For example, horizontal ellipsoid represents transcription regulators and vertical ellipsoid represents transmembrane receptor. Solid lines represent direction interaction and dashed ones indirect interaction. A detailed tutorial can be referred to webpage <http://ingenuity.force.com/ipa/articles/Feature_Description/Legend>.


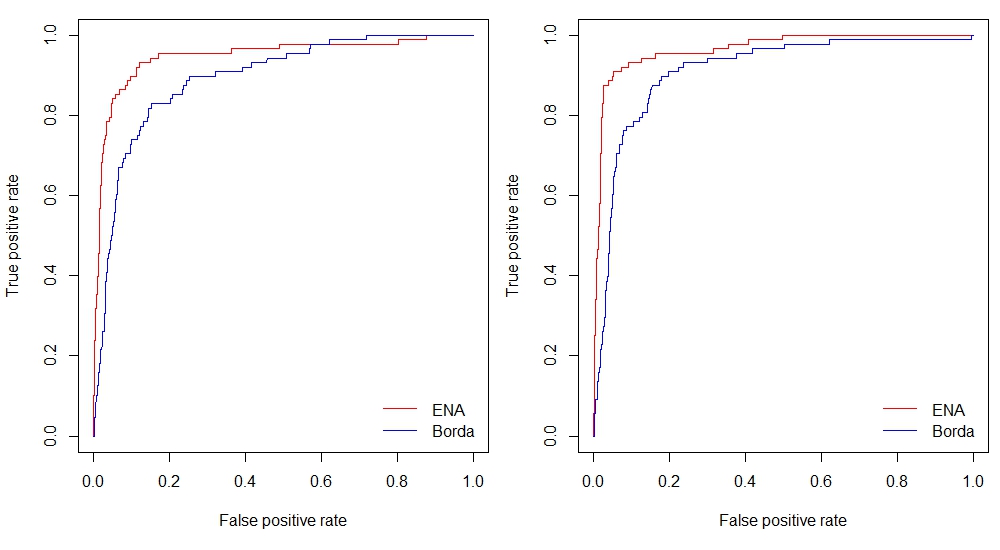


Figure S8. The comparison between the ENA approach and the Borda count method. A comparison of the accuracy of the reconstructed networks using the datasets containing 200 samples (left) and 1,000 samples (right) from the 83-gene network with a noise value of 0.25. When we used ENA and Borda count method (used by DREAM5 challenge, Marbach, et al.) to integrate SPACE, WGCNA and GeneNet, ENA performed better than the Borda method. AUC (Left, ENA: 0.947, Borda: 0.893; Right, ENA: 0.966, Borda: 0.911)
